# Understanding Mental Health in the Context of Adolescent Pregnancy and HIV in Sub-Saharan Africa: A Systematic Review Identifying a Critical Evidence Gap

**DOI:** 10.1007/s10461-020-03138-z

**Published:** 2021-01-15

**Authors:** Kathryn J. Roberts, Colette Smith, Lucie Cluver, Elona Toska, Lorraine Sherr

**Affiliations:** 1 Institute for Global Health, University College London, London, UK; 2 Department of Social Policy and Intervention, University of Oxford, Oxford, UK; 3 Department of Psychiatry and Mental Health, University of Cape Town, Cape Town, South Africa; 4 Centre for Social Science Research, University of Cape Town, Cape Town, South Africa; 5 Department of Sociology, University of Cape Town, Cape Town, South Africa

**Keywords:** Adolescent pregnancy, Adolescent parenthood, HIV/AIDS, Common mental disorder, Mental health, Sub-Saharan Africa

## Abstract

Adolescent (10–19 years) mental health remains an overlooked global health issue. Rates of adolescent pregnancy within sub-Saharan Africa are some of the highest in the world and occur at the epicentre of the global HIV epidemic. Both experiencing adolescent pregnancy and living with HIV have been found to be associated with adverse mental health outcomes, when investigated separately. Poor mental health may have implications for both parent and child. The literature regarding mental health within groups experiencing both HIV and adolescent pregnancy is yet to be summarised. This systematic review sought to identify (1) the prevalence/occurrence of common mental disorder amongst adolescents who are living with HIV and have experienced pregnancy, (inclusive of adolescent fathers) in sub-Saharan Africa (2) risk and protective factors for common mental disorder among this group, and (3) interventions (prevention/treatment) for common mental disorder among this group. A systematic search of electronic databases using pre-defined search terms, supplemented by hand-searching, was undertaken in September 2020. One author and an independent researcher completed a title and abstract screening of results from the search. A full-text search of all seemingly relevant manuscripts (both quantitative and qualitative) was undertaken and data extracted using pre-determined criteria. A narrative synthesis of included studies is provided. Quality and risk of bias within included studies was assessed using the Newcastle-Ottawa scale. A systematic keyword search of databases and follow-up hand searching identified 2287 unique records. Of these, thirty-eight full-text quantitative records and seven full-text qualitative records were assessed for eligibility. No qualitative records met the eligibility criteria for inclusion within the review. One quantitative record was identified for inclusion. This study reported on depressive symptomology amongst 14 pregnant adolescents living with HIV in Kenya, identifying a prevalence of 92.9%. This included study did not meet the high methodological quality of this review. No studies were identified reporting on risk and protective factors for common mental disorder, and no studies were found identifying any specific interventions for common mental disorder for this group, either for prevention or for treatment. The limited data identified within this review provides no good quality evidence relating to the prevalence of common mental disorder among adolescents living with HIV who have experienced pregnancy in sub-Saharan Africa. No data was available relating to risk and protective factors or interventions for psychological distress amongst this group. This systematic review identifies a need for rigorous evidence regarding the mental health of pregnant and parenting adolescents living with HIV, and calls for granular interrogation of existing data to further our understanding of the needs of this group. The absence of research on this topic (both quantitative and qualitative) is a critical evidence gap, limiting evidence-based policy and programming responses, as well as regional development opportunities.

## Introduction

Adolescent (10–19 years) [1] mental health remains an overlooked global health issue [2–5]. Fifty percent of mental health disorders are established by 14 years of age and, 75% before 24 years of age [6]. Poor mental health within adolescence has implications across the life course; affecting both physical and mental morbidity [5–11]. At a societal level, poor mental health has widespread socioeconomic impacts both within the short and long term; having implications for workforce participation, healthcare systems, and, economic growth [12–14]. Mental health disorders are a leading cause of disability, affecting an estimated 10–20% of children and adolescents globally [2, 15]. However, estimates may not be wholly representative as the coverage of prevalence data for mental disorders among children and adolescents remains limited, particularly within low and middle income countries; potentially skewing global estimates [16–19]. Few studies relating to the treatment and prevention of mental health have targeted children and adolescents, and those that do often focus on developmental disability rather than broader mental disorder [19]. Africa is home to the fastest growing adolescent population in the world, predicted to reach 435 million by 2050 [20]. Promoting adolescent potential, of which mental health is a fundamental aspect, is critical to the success and prosperity of both the individual and the region. A stronger understanding of mental health is required to inform policy and programming to promote adolescent potential. This increased understanding is particularly important when compounded by other comorbidities or syndemic conditions; as the presence of multiple phenomena may have implications for the assessment, treatment and experience of mental disorders.

Rates of adolescent pregnancy (10–19 years) within sub-Saharan Africa are some of the highest in the world (almost one in five (~20%) female adolescents experience pregnancy between 10 and 19 years of age) [21]. These high rates of adolescent pregnancy occur within the epicentre of the global HIV epidemic (sub-Saharan Africa is home to ~1.5 million adolescents living with HIV) [22, 23]. Poor mental health has been found to be prevalent among adolescents living with HIV [24, 25] and, likewise, those experiencing adolescent pregnancy [26, 27] within separate explorations. In addition to navigating the developmental period of adolescence, parenting adolescents (both mothers and fathers) are also navigating pregnancy, childbirth and childrearing. For adolescents living with HIV, there are additional considerations such as health, stigma, HIV transmission (both perinatal and postnatal), and adherence to medication [28]. The dual impact of such phenomena may compound experiences of mental disorder. Mental health may have a bidirectional relationship with the experience of adolescent pregnancy and HIV [26, 29, 30]. For example, worse mental health may lead to increased risk behaviour (i.e. unprotected sex), which may in turn result in adolescent pregnancy and/or postnatal HIV infection. Likewise, adolescent pregnancy and/or living with HIV may contribute mental health challenges. Parental mental health has been found to impact development outcomes for children [27, 31–34]. It is important to understand whether parental mental health in the presence of HIV and adolescent pregnancy is similarly problematic. The literature regarding mental disorder within groups experiencing both phenomena is yet to be summarised. Due to the prevalence of both HIV and adolescent pregnancy within the sub-Saharan African region, a large sub-group are living with HIV, and have experienced pregnancy. Taking into account the current global estimates of mental disorder among children and adolescents, a large proportion of this population may be experiencing poor mental health.

Commonly emerging mental health challenges within adolescence comprise emotional disorders including depression and anxiety. Globally, depression and anxiety are leading causes of illness and disability among adolescents [35, 36]. Such disorders have profound impacts; affecting daily life and, at their worse have implications for mortality (i.e. depression may lead to suicide) [35, 37]. Within contexts of high poverty, high violence, high communicable disease (i.e. HIV) and high levels of adolescent childbirth, such as within sub-Saharan Africa, trauma may also present challenges for adolescents [38–41]. Due to the potentially commonality of such mental health challenges among adolescents (including the population of interest), and the emergence of transdiagnostic approaches to interventions for mental health among adolescents within low and middle income countries [42], for the purpose of this review depression, anxiety, trauma and suicidality are subsumed under the rubric of ***common mental disorder*** [43]. Such disorders will additionally be explored separately where possible. The concept of common mental disorder has been used extensively within the global mental health field [5, 43–45]. In recent years, there has been a call to shift away from binary classifications of mental disorder towards a continuum approach to mental health to better reflect the diversity and complexity of mental health experience within the global mental health field [44, 46]. However, this is yet to be implemented at scale [46]. Due to the potential paucity of research focusing on adolescent mental health experience within sub-Saharan Africa, the use of existing classification systems seemingly will provide the most substantial evidence in relation to mental health experience at this time. While it is important not to pathologise or reduce the contextual understanding of the mental health experience of adolescents through such labelling, it is critical that need in relation to mental health experience and elevated poor mental health symptomology is identified to ensure that adequate and effective support can be provided.

Summarising available data is critical to assessing the scale of common mental disorder among adolescents living with HIV who have experienced pregnancy (inclusive of adolescent fathers), enhancing our understanding regarding the mechanism of effect for common mental disorder, and to establishing an awareness of effective interventions for common mental disorder, should such interventions be required. Such a summary is imperative to developing understanding regarding the needs of adolescent parents living with HIV, to establish where gaps in evidence remain, and is fundamental in guiding future research directives. Addressing common mental disorders within adolescence, and particularly for this sub-group, is imperative to promoting the fulfilment of potential for not only adolescents themselves but for their children also, and as such, future generations.

This is the first systematic review to summarise the evidence regarding mental health among adolescents who have experienced pregnancy (those who are currently pregnant or experienced pregnancy during adolescence [inclusive of adolescent fathers]) and are living with HIV in sub-Saharan Africa. Specific objectives of this review were to:

1. identify the prevalence/occurrence of common mental disorder amongst adolescents living with HIV experiencing pregnancy/fatherhood (10–19 years old) in sub-Saharan Africa
2. identify risk and protective factors for common mental disorder amongst adolescents living with HIV experiencing pregnancy/fatherhood (10–19 years old) in sub-Saharan Africa
3. identify which psychosocial interventions have aimed to reduce common mental disorder amongst adolescents living with HIV experiencing pregnancy/fatherhood (10–19 years old) in sub-Saharan Africa, and the effectiveness of such interventions.

## Methods

This systematic review adheres to the *Preferred Reporting Items for Systematic Reviews and Meta-Analyses* (PRISMA) guidelines [47]. Prior to commencement, the protocol for this systematic review was registered via the PROSPERO database (protocol number: CRD42019133585). To provide a comprehensive overview of mental health with the context of adolescent pregnancy and HIV, both quantitative and qualitative studies were included within this review.

## Inclusion and Exclusion Criteria

Those studies meeting the pre-specified inclusion criteria and none of the exclusion criteria were included in the review findings. See Table 1 for the inclusion and exclusion criteria used within this review. Studies were included if the outcomes of interest (*common mental disorder* [for quantitative studies]/*mental health experience* [for qualitative studies]) were clearly identifiable for the population of interest (adolescents living with HIV who had experienced pregnancy i.e. currently pregnant adolescents, or individuals who experienced pregnancy during adolescence [inclusive of adolescent fathers]). Quantitative studies utilising outcome measures relating to common mental disorder were identified for inclusion. Common mental disorder is often used as an overarching term within the field of mental health and encompasses an array of mental health burdens [5, 43–45]. Many validated measures relating mental health are often labelled with reference to common mental disorder i.e. the Shona Symptom Questionnaire (a measure of common mental disorder) [48]. However, the concept of common mental disorder is much broader, [5, 43–45] and therefore search terms were extended to map additional concepts inclusive of depression, anxiety, trauma, and suicidality to identify studies utilising such measures from which we could glean data relating to prevalence, predictors, and interventions for mental health among the group of interest. Within the identification of qualitative studies, the outcome measures of interest were expanded to include any discussion of experience relating to mental health among the population of interest to glean the most information relating to the population of interest.

**Table 1:** Inclusion and exclusion criteria for studies within this systematic review

	Inclusion criteria		Exclusion criteria
	Quantitative studies	Qualitative studies	
Population	Individuals living with HIV who are currently or have previously experienced pregnancy during adolescence (10–19 years; inclusive of adolescent fathers)^a^		
Outcome measure	− Validated measures of common mental disorder inclusive of depression, anxiety, PTSD, suicidality and/or self-harm	− Any exploration of mental health experience as defined by the study	
Geographical location	Sub-Saharan Africa (see list of included countries in Fig. 1.		Studies undertaken wholly outside of sub-Saharan Africa
Study design	− Randomised control trials − Cluster randomised control trials − Quasi experimental studied with an identified control group − Controlled before and after studies − Longitudinal cohort studies − Cross-sectional studies (prevalence only)	− Qualitative studies	
Publication type	− Peer reviewed manuscripts − Grey literature including dissertations and theses − Conference abstracts and presentations with sufficient information		− Books
Language	English, French		All other languages
Intervention (for relevant studies only)	Psychosocial; psychological (i.e. psychological therapies), psychosocial (i.e. care, playgroups, social support, school-based interventions), physical (i.e. medical interventions), economic (i.e. economic assistance, material assistance).		

^a^To be included within the review, data should disaggregate by HIV and pregnancy status

## Search Strategy and Selection Criteria

**Fig. 1:**
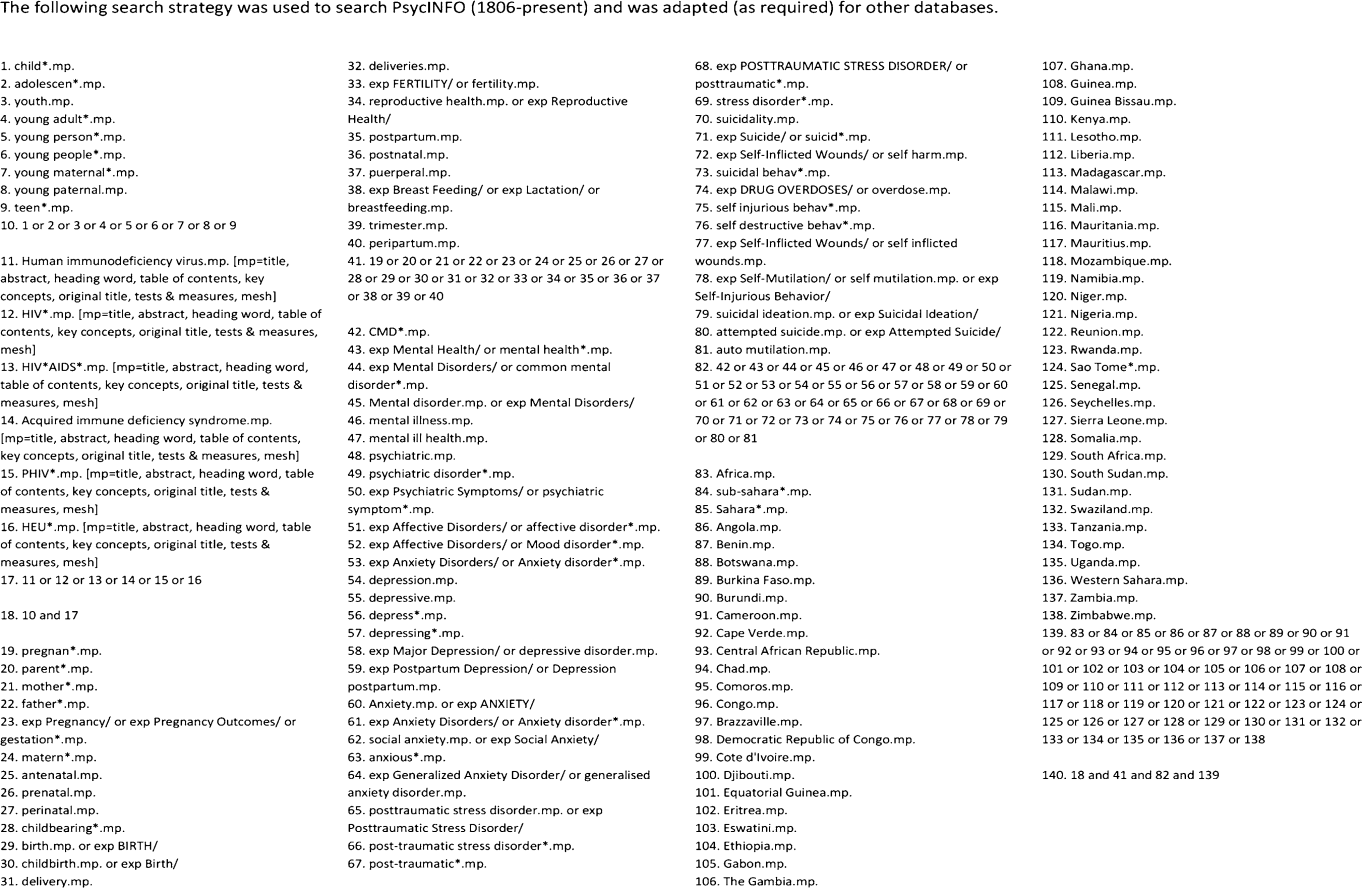
Search strategy

Using a pre-determined search strategy, studies considered for inclusion within this review were identified through a systematic search of electronic bibliographic databases. Databases searched included: PsycINFO (1806-present), PsycExtra (1900-present), PsycARTICLES, Embase, PubMed (including MEDLINE, 1966-present), Global Health Archive (1910–1972), Web of Science (1900-present) and, the Cochrane database of systematic reviews. Campbell Collaboration Records and PROSPERO register for systematic reviews were also hand searched using multiple variations of the search terms used within this review (see Fig. 1 for a list search terms used within the review). Citations within manuscripts selected for full-text review were also used to identify works for inclusion within this systematic review. Database searches were conducted in September 2020.

## Selection of Studies and Data Extraction

All titles and abstracts identified through electronic database searching and hand searching were examined for relevance. Full manuscripts of any potentially relevant studies were obtained and assessed for inclusion, based on the above inclusion criteria. Where uncertainty arose within the examination process, full manuscripts were acquired for further scrutiny. An independent researcher, for validation, reviewed a subsection of identified titles and abstracts (10%)—no discrepancies arose between the lead author and the independent researcher. Data from studies that met the inclusion criteria were extracted using a standardised extraction form. Data extraction was carried out by the lead author (KJR) and, scrutinised by the wider writing team. No cases of disagreement arose. Information extracted from relevant manuscripts included publication detail, study methodology, geographical location, sample details, details of measures and relevant findings.

## Assessment of Quality and Risk of Bias of Included Studies

As only non-randomised quantitative studies were included within this review, the Newcastle-Ottawa scale was used to assess risk of bias based on the recommended practice of the Cochrane Collaboration [49, 50]. The Newcastle-Ottawa scale assesses the quality of the evidence within this review based on selection methods of the study i.e. representativeness, the comparability of groups of interest and the quality of the outcome measure. The scale ranges from 0 to 9, with scores ≥6 indicative of methodological quality [50]. To maximise the validity of the assessment, both the lead author (KJR) and an independent researcher completed the assessment for included studies. No disagreement arose between the two reviewers.

## Data Synthesis

A narrative synthesis providing a descriptive summary of studies meeting the inclusion criteria of the review is presented.

## Results

**Fig. 2:**
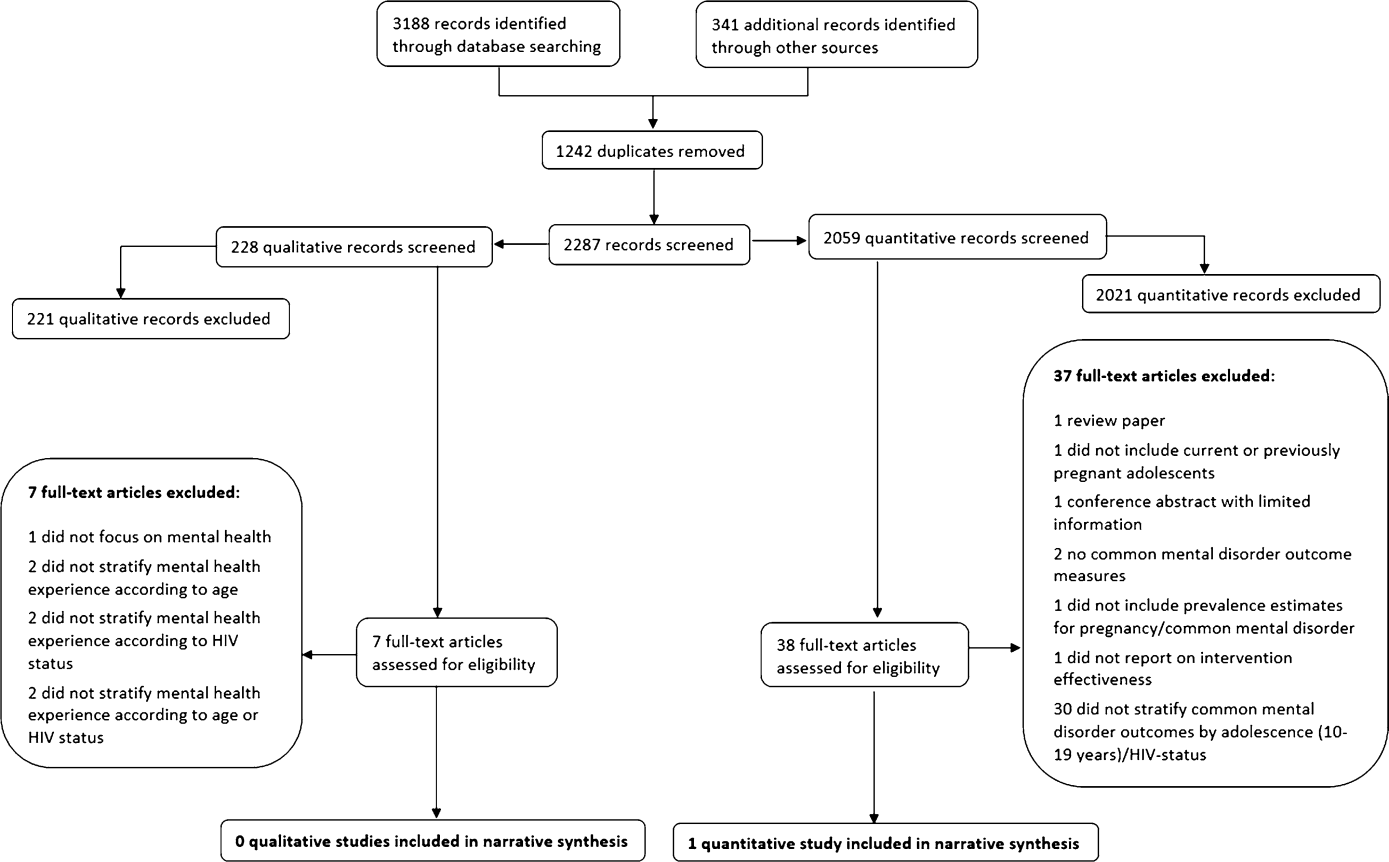
Study flow diagram

Following the removal of duplicate records, 2287 citations were identified from electronic databases and hand searching; 2059 quantitative studies and 228 qualitative studies. After completing title and abstract screens of all 2287 citations, inclusive of full-text screens of 38 quantitative records and seven qualitative records, one quantitative manuscript was eligible for inclusion based on the first objective of this review. No qualitative records were identified which met the inclusion criteria for the review (see Fig. 2).

This single study, undertaken in Kenya, focuses on a study sample of pregnant female adolescents and stratifies data relating to a positive depression screen according to HIV status as part of sub-analyses (see Table 2). Fourteen pregnant adolescents living with HIV were included within the study. Amongst those 14, 13 (92.9%) screened positive for symptomology consistent with depression whereas, 27.8% (45/162) of those not living with HIV in the sample screened positive for symptomology consistent with depression. Pregnant adolescents living with HIV in the sample also reported higher mean depressive symptomology scores comparative to pregnant adolescents not living with HIV (17.4 vs. 10.6, respectively; PHQ-9 scored 0–27). While an exploration of risk factors for common mental disorder was undertaken relating to the overall sample within this study, such findings were not stratified accord to HIV status and were therefore not included within this review [51].

**Table 2:** Studies identified for inclusion within the review

Author and year	Country	Study design	Measure of CMD	Total sample (n)	Adolescents living with HIV (n)	Age	Female (%)	Male (%)	Study population	Prevalence
Osok et al. (2018) [51]	Kenya	Cross-sectional survey	Depression; Patient Health Questionnaire-9	176	14 (8.0% total sample)	15–18 years	100%	n/a	Currently pregnant female adolescents recruited from an antenatal care clinic	Prevalence of depression amongst adolescents living with HIV was found to be 92.9%

No manuscripts were identified which met the criteria for inclusion for objectives 2 (identifying risk and protective factors for common mental disorder) or 3 (interventions for common mental disorder) of this review.

## Quality of Included Studies and Risk of Bias

Table 3 provides a summary of the methodological quality of the singe study identified by this review [51]. Overall, the study was given a score of 2 (scored 0–9), indicative of poor methodological quality due to multiple sources of bias (including: the representativeness of the sample, the ascertainment of pregnancy and combined HIV status, an inability to demonstrate that depressive symptomology was not present prior to pregnancy and combined HIV status, the use of self-reported outcome measures to ascertain depressive symptomology, and limitations directly linked to the use of cross-sectional data inclusive of a lack of follow-up data). It should be noted that prevalence data (relating to objective 1 of this review) by definition is cross-sectional.

**Table 3:** Quality assessment of studies included within this review (Osok et al. 2018) [51]

Newcastle-Ottawa Scale (NOS) of methodological quality									
Selection				Comparability	Outcome			NOS total score (0–9)	Overall methodological quality
Representativeness of cohort	Selection of non-exposed cohort	Ascertainment of exposure	Presence of outcome of interest at study initiation	Comparability of cohort	Assessment of outcome	Length of follow-up	Adequacy of follow-up		
0	1	0	0	1	0	N/A	N/A	2	Poor

## Discussion

Within this review, only one quantitative study was identified that commented on the prevalence of common mental disorder amongst pregnant adolescents living with HIV in sub-Saharan Africa. Within the identified study, the prevalence of depressive symptoms among pregnant adolescents living with HIV (a sub-sample of the whole sample included within the study; *n* = 14) was 92.9% [51]. No studies were identified reporting on risk and protective factors for mental health and, no studies were found identifying specific interventions for mental health for this group. The study included within this review should be commended for the inclusion of disaggregated data relating to adolescents living with HIV who have experienced pregnancy. However, any inferences drawn from this data are limited, as the study did not meet the high methodological quality requirements set out in this review.

While only one of the identified studies met the inclusion criteria for this review, numerous quantitative studies were identified within the search as partially relevant to the review. These studies did not meet the inclusion criteria as they did not stratify common mental disorder outcomes by age (adolescence; 10–19 years) or HIV status. These studies focusing on pregnancy and parenthood, (possibly including adolescents living with HIV based on the sampling strategy), may contribute insight into the mental health experience of pregnant (currently or previous) adolescents living with HIV. One study documenting the experience of adolescents living with HIV (male and female) reported the unadjusted association between ever being pregnant and current mental health status, and identified no difference in mental health scores relating to experience of pregnancy. This study was not included within the review, as it did not report on the number of participants within the study who reported pregnancy. Therefore, prevalence of common mental disorder for this specific sub-group could not be ascertained [52].

In relation to prevalence of common mental disorder, 15 studies reporting on common mental disorder considered for inclusion within this review did include pregnant or previously pregnant adolescents within their samples [53–67]. However, 11 of these studies did not stratify outcomes of common mental disorder according to age, so prevalence rates within the adolescent group could not be identified [53–55, 57, 58, 60–63, 66, 67]. Four studies did stratify common mental disorder outcomes according to adolescence, yet did not stratify by HIV status [56, 59, 64, 65]. Within these studies, rates of common mental disorder amongst pregnant adolescents (two studies focused on pregnancy [59, 64], one the postpartum period [0–36 months] [65] and, one on both pregnancy and the postpartum period [56]) ranged from 8.8% to 21.6% (total number of pregnant/previously pregnant adolescents ≥14 years included within the studies = 517) [56, 59, 64, 65].

Four additional studies that included adolescents within the larger study sample (but did not stratify according to age or HIV status) reported on factors associated with common mental disorder. Post-partum depression was found to be associated with impaired child growth [56]. Psychological morbidity during pregnancy was not associated with age, employment status [59] nor HIV [59, 64]. Psychological morbidity among mothers was associated with having an older spouse in one study in Zimbabwe [64], and, experiencing verbal/physical abuse, having a partner who did not help with childcare, being in a polygamous relationship, having a partner with lower levels of education and, having a partner who smoked in a study in Tanzania [65]. Furthermore, a study undertaken in Kenya, identified younger age, experience of a stressful life event and living with HIV as being associated with increased depressive symptomology during pregnancy, and social support as being a protective factor for depression within this period. While this study was included within the review in relation to prevalence, the examination of factors associated with depressive symptoms did not stratify by adolescents living with HIV [51].

A further five studies were identified which were likely to have included adolescents within their sample based on the reported ages of the sample (presented as an average, alongside a measure of variability) reported on factors associated within common mental disorder. Within these five studies, common mental disorder was found to be associated with economic difficulties [68, 69], relationship difficulties [69], HIV infection [69], infant health issues [69, 70], food insecurity [71], and experience of intimate partner violence [68].

A further six studies included populations aged ≥18 years [72–77]. As such, some older adolescents (18–19 years) may have been included within the sample. Within these studies, antenatal depression was found to be associated with unintended pregnancy [76] and younger age [72] (amongst pregnant women living with HIV), intimate partner violence [73, 77] and, previous history of depression [77].

Populations of ever-pregnant adolescents (not stratified by the adolescent period) were included in two studies (population age: 18+ years [75] and 14–46 years [60]), which stratified mental health outcomes by HIV status. These studies report mixed results. One study identified HIV as being associated with depressive symptoms [60] and, one reported depressive symptoms to be more prevalent amongst those participants who were known to be HIV-negative in the sample when compared to those who were living with HIV [75].

Likewise, six studies (which included, or may have included adolescents living with HIV within their sample [due to a lack of specific information regarding age and/or HIV status within the sample]) focusing on the associations between common mental disorder amongst mothers, and child outcomes report mixed results. Three studies identified no difference in child outcomes according to common mental disorder status [54, 57, 68]. However, three studies identified common mental disorder as having a negative effect on child behaviour [61], child growth [56, 74], child emotional development and peer problems [74]. In a longitudinal sample of 70 mothers living with HIV, aged 16–64 years, the presence of common mental disorder was found to be associated with worse child behaviour outcomes [61].

In relation to interventions, a study undertaken in Uganda reported on the development of a community-based intervention aimed at improving the wellbeing of adolescent mothers. However, it was not clear if adolescent mothers were living with HIV, and no results relating to intervention effects were reported [78]. Two intervention studies in which participants either received visitation from community health workers or standard care were undertaken within South Africa were identified. The samples for these studies likely included a small number of adolescents living with HIV who had experienced pregnancy, however, results were not disaggregated accordingly. The interventions provided mixed results. When undertaken in a peri-urban area in which participants received visits from community health workers, the intervention was found to reduce maternal depression at 6-month follow-up when compared standard available care [79]. However, a similar intervention, undertaken in a rural area, found no significant differences regarding maternal depression scores between groups in the first 2 years postpartum [80].

Within the search, a single study identified the rate of adolescent fatherhood to be 11.1% in South Africa. This study included data on common mental disorder as well as data on adolescent pregnancy and adolescent fatherhood. However, data regarding common mental disorder was not stratified by parenthood status and, data regarding HIV status was not reported [66].

Despite no qualitative studies being identified relating to mental health within the population of interest, several studies were identified which gave a partial view of the mental health experience of this group. While adolescents living with HIV who had experienced pregnancy (10–19 years) were included within two studies (total *n* = 12; two articles were drawn from the same dataset) undertaken in South Africa [81–83], findings relating to mental health were not disaggregated by age (adolescence; 10–19 years at time of pregnancy) [82, 83] or HIV status [81]. While it was not possible to identify prevalence of common mental disorder from qualitative studies, descriptive experience of, and themes/experiences linked to poor mental health/emotionality were identified. Studies described “*despair*” and “*sadness*” related to pregnancy discovery [82], “*anger*”, “*embarrassment*” and “*suicidal ideation*” related to pregnancy, “*guilt*” related HIV discovery and, a “*fear*” of transmitting HIV to their unborn child [83]. “*Stress*” and “*anxiety*” were found to be linked to pregnancy disclosure rather than HIV disclosure, a withdrawal from schooling and, unstable family and partner relationships following pregnancy discovery. Adolescents also described “*sadness*” related to HIV discovery, a lack of partner support postpartum and, feeling that the baby had become a burden to their family [81]. Much of the data related to the pregnancy period and pregnancy discovery, with little mention of the postpartum or parenting period [81–83]. Themes related to poor mental health within this context were feelings of unpreparedness relating to imminent motherhood and limited financial and emotional support [82, 83]. These studies identified the female adolescents’ families, particularly the maternal grandmother of the child, as critical in providing financial, emotional, psychological and physical support. Such support was identified as a key feature of “*coping*” [81–83]. Mental health experience does not remain static and as such may change over time. As such, these studies are limited by their focus on the period relating to pregnancy discovery as broader experiences relating to pregnancy, post-partum and parenting periods may be better placed to inform policy.

Adolescents living with HIV who had experienced pregnancy were or were likely to be included within a further three qualitative manuscripts based on information relating to the study sample however, results and emerging themes were again not disaggregated by age or HIV status [84–86]. These studies focused on experiences of “*depression*”, “*suicidal ideation*” [84–86] and “*anxiety*”; [85] some of which mapped on to clinical CMD symptomology [85]. Potential themes identified as being linked poor mental health experiences were intimate partner violence [84], lack of social support [84–86], social isolation and stigmatisation related to both HIV and pregnancy [84, 85], poverty and a lack of material support [84–86] and child illness [85]. Within secondary analyses, one study highlighted the interconnected and often bidirectional relationship between early (adolescent) pregnancy, HIV, and mental health, and emerging importance of mental health within their original study focusing on HIV-affected female adolescents [86].

A single qualitative study focused on a peer mentoring intervention for adolescents living with HIV who had experienced pregnancy relating to PMTCT. While mental health was not a focus of the intervention, the authors identified loneliness as being linked to anxiety among the study population and a desire for receiving psychosocial support from adolescent peer mentors; highlighting an example of an active intervention that may be a vehicle for psychosocial support for this population [87]. None of the qualitative studies identified which offered a partial view of the mental health experience of adolescents living with HIV who experienced pregnancy focused on adolescent fathers.

## Adolescent Pregnancy, HIV, and Mental Health: A Critical Evidence Gap

Disregarding attempts to supplement our understanding of the mental health experience of pregnant adolescents living with HIV with partially relevant studies, this systematic review identified a single study focusing on common mental disorder among pregnant adolescents living with HIV (*n* = 14) in Kenya. As such, mental health need among this group remains underexplored, and there remains an absence of data from the sub-Saharan African region. Within this review, and among the majority of studies identified deemed relevant to this review, the measures of common mental disorder relate to depressive symptomology, with few also commenting on measures of overall common mental disorder. Hence, there remains a limited understanding of other common mental disorder outcomes (i.e. anxiety, trauma, suicidality) and the impacts of such experiences for both adolescents and their child(ren).

While a single prevalence estimate of depressive symptomology was identified, no exploration of risk and protective factors for common mental health nor, interventions to prevent or treat common mental disorder were identified for this group—despite evidence that pregnant adolescents (potentially living with HIV) are seemingly accessing some support [78]. As such, the effectiveness of any interventions currently in place is unknown. This finding supports previous research highlighting a lack of prevention interventions targeting mental health among adolescent parents within low and middle income countries [88]. Furthermore, no studies exploring the mental health experience and needs of adolescent fathers living with HIV were identified. Adolescent mental health experiences have been found to have adverse outcomes across the life course [5–11]. For pregnant adolescents (current or previously), poor mental health has been found to have adverse impacts for both parent and child (i.e. bonding, child development) [27]. For adolescents living with HIV, poor mental health has been found to have negative implications for service engagement, medication adherence and risk behaviours [28]. Hence, for pregnant adolescents living with HIV, additional considerations may arise; poor mental health may have impacts on service engagement (HIV, sexual and reproductive health, antenatal) and onwards HIV transmission (i.e. prevention of mother to child transmission)—concerns critical to both adolescent and child wellbeing [89]. Adolescents living with HIV (both mothers and fathers) and their children may have specific needs, and therefore, further investigations into the mental health needs of this group are required to bolster positive outcomes for this group.

There is seemingly a focus within the available literature on the pregnancy period with less attention given to the post-partum period and beyond. An exploration of outcomes for children born to adolescents living within HIV in relation to adolescent mental health also remains absent. Data for adult populations who may have experienced adolescent pregnancy may exist. However, a lack of stratification, results in the mental health experience of adolescents living with HIV as they progress into adulthood and the subsequent impacts for their children, again, being absent from literature. Common mental disorder is not a static phenomenon, therefore mapping mental health experience over the life course is critical to assess how mental health need may change, and at which stage/under which circumstances intervention should be targeted.

The period of adolescence bridges childhood and adulthood and as such, data regarding this critical developmental stage is not consistently presented within the literature and may be missed within the presentation of data. Focused data on the adolescent period is urgently required. Seemingly, data may be available regarding adolescent pregnancy and parenthood within the context of HIV within existing studies and databases. Many researchers only include individuals over 18 years of age due to ethical concerns, more stringent ethical review processes and the challenges of engaging children and adolescents under 18 years old (i.e. logistical challenges, the requirement for specifically trained researchers and/or data collectors and the need for age-appropriate measures and study materials). However, not reporting the needs of adolescents—a potentially vulnerable group—also raises ethical concerns, as a lack of evidence may result in inadequate policy, programming and resource allocation.

There is a dearth of knowledge regarding common mental disorder among currently or previously pregnant adolescents (inclusive of adolescent fathers) living with HIV in sub-Saharan Africa. This data gap is seemingly not simply that data regarding this group is not collected—data exists relating to common mental disorder within adolescent pregnancy and, likewise adolescents living with HIV—but partially because data is not disaggregated by age, HIV status, pregnancy/parenthood status. As such, this data becomes lost within the findings relating to broader groups. Clustering data regarding adolescents with young adults (i.e. 15–24 years) remains a common feature within international data collection strategy [90, 91], and often adolescents are described as adult women within data and research following the birth of a child (possibly due to the legal emancipation of adolescent mothers within some countries, i.e. Kenya) [92]. Such practices distort our understanding of the experience of adolescents living with HIV who have experienced pregnancy. This group should not be forgotten within research and policy agendas. This study echoes and extends calls for more granular data regarding adolescents living with HIV [93], and high quality data regarding interventions for the prevention and treatment of mental health in adolescents living with HIV [94]. Expanding the scope of inquiry to include more explicit and scrupulous examination of the mental health needs of pregnant adolescent living with HIV is essential to ensuring that this adolescent group and their children are able to reach their potential. Disaggregation of available data would be a prompt and valuable exercise to better our understanding of this group, and to inform policy and programming. However, methodologies should also remain a consideration within future investigations relating to this group to ensure robust data to better inform policy where possible, inclusive of collecting data regarding pregnancy/parenthood, the use of longitudinal data, and greater access and analyses of available clinical data. The mental health of pregnant adolescents has socioeconomic implications for individuals, communities and wider society. Ensuring adolescent mental health—inclusive of this potentially vulnerable group—remains at the core of development agendas is critical to providing the building blocks of prosperity for future generations and societies. Yet, to do this, an evidence base is required. At this time, this evidence base is lacking.

## Limitations

The findings from this review should be interpreted in the context of study limitations. Firstly, while extensive efforts were made to include a wide range of databases, not all were included. Likewise, grey (unpublished) literature was not included within this review. As such, some relevant studies may have been overlooked within the identification stage of this review. Secondly, some studies which may have included samples aged 18 years+ (older adolescents) focusing on common mental disorder, pregnancy, and HIV may not have been included within the identification process of this review, as the search terms within this review did not focus on adult populations. Thirdly, due to resource constraints, only manuscripts written in the English or French language (due to the high prevalence of French speakers within sub-Saharan Africa) were included within the review. Fourthly, it should be noted that the use of terminology within this review i.e. *common mental disorder* and the dependence on validated screening tools within the inclusion criteria for quantitative studies may limit investigation into broader experience of mental health for adolescents living with HIV who have experienced pregnancy within sub-Saharan Africa. While the use of such measures and classifications hold clinical utility [44, 46], individuals who may be experiencing some dimension of poor mental health but do not reach the clinical threshold may be missed within investigations. While there is movement within the field of global mental health towards broader classifications of mental health experience [44, 46], such detail is often not included within current clinical, research, or policy practice within sub-Saharan Africa [46]. Hence, the utilised classifications were deemed the most relevant in relation to the current state of the evidence, the most appropriate for summarising the experience of the population of interest in a timely manner, and the most accessible to policymakers. In an attempt to incorporate broader mental health experiences, qualitative studies relating to mental health focusing on the population of interest were additionally included as part of this review however, no manuscripts were identified focusing on the population of interest. The contextual, conceptual, and linguistic relevance of such measures to adolescents within sub-Saharan Africa should also be noted [86]. The clinical interpretation of disorders such as depression, anxiety, trauma, or suicidality may not be captured within adolescents’ articulation of such experiences [86, 95]. Likewise, often these disorders do not have direct linguistic translation within many languages used across sub-Saharan Africa potentially restricting the interpretation of diagnostic terminology. However, while recent research has identified some confusion among adolescents within sub-Saharan Africa over terminology definitions relating to mental health i.e. *anxiety*, items within clinical measures of mental health screening tools have been found to be relevant and understood among adolescents experiencing pregnancy within sub-Saharan Africa [96]. Finally, it was beyond the scope of this review to explore the common mental disorder among pregnant (currently or previous) adolescents not living with HIV. Pregnant adolescents have been found to be at a greater risk of postnatal HIV infection [97–99], which may in turn have implications for mental health. Therefore, this group should not be forgotten within the research or programming response.

## Conclusions

Good mental health is critical to allow adolescents and their child(ren) to reach their full potential and thus, in turn, the success of the individuals, and at a societal level within the sub-Saharan African region. Yet, the mental health of pregnant adolescents (currently or previously) living with HIV (including both mothers and fathers) within sub-Saharan Africa has been neglected within the literature. As such, there is an absence of knowledge regarding the prevalence of common mental disorder amongst this group, risk and protective factors for common mental disorder and the effectiveness of interventions—limiting evidence-based policy and programming for this group. This systematic review identifies a need for rigorous evidence regarding the mental health of pregnant adolescents living with HIV, and calls for granular interrogation of existing data to further our understanding of the needs of this group. There remains a pressing need to explore the mental health experience of pregnant adolescents living with HIV (inclusive of antenatal experience, post-partum and fatherhood), and to assess the effectiveness of existing interventions being implemented for common mental disorder among pregnant (currently or previously) adolescents living with HIV.
